# Safety and satisfaction of patients with nurse's care in the perioperative*

**DOI:** 10.1590/1518-8345.2646.3142

**Published:** 2019-04-29

**Authors:** Amalia Sillero-Sillero, Adelaida Zabalegui

**Affiliations:** 1 Hospital de la Santa Creu i Sant Pau, Barcelona, Espanha; 2 Hospital Clinic of Barcelona, Barcelona, Espanha

**Keywords:** Perioperative Nursing, Patient Safety, Patient Satisfaction, Adverse Events, Perioperative Care, Health Facility Environment, Enfermagem Perioperatória, Segurança do Paciente, Satisfação do Paciente, Eventos Adversos, Cuidados Perioperatórios, Ambiente de Instituições de Saúde, Enfermería Perioperatoria, Seguridad del Paciente, Satisfacción del Paciente, Eventos Adversos, Cuidados Perioperatorios, Ambiente de Instituciones de Salud

## Abstract

**Objective:**

to investigate the safety and satisfaction of patients and their relationship with nurse's care in the perioperative period.

**Method:**

cross-sectional, multi-level, correlational study with 105 nurses in the surgical area and 150 patients operated in a Spanish tertiary hospital. For the nurses the sociodemographic variables, the perception of the work environment, the professional burnout and the satisfaction in the work were collected. For patients, the safety of adverse events and level of satisfaction, through the application of questionnaires. Univariate and multivariate analysis were used.

**Results:**

job satisfaction, professional commitment, and participation in hospital issues were negative predictors for adverse events related to the patient, while postoperative nurse care was a positive predictor.

**Conclusion:**

there is an increase in adverse events when nurses are dissatisfied at work, less professional commitment and low availability to participate in the subjects of their unit. On the other hand, adverse events decrease when nurses perform the care in the postoperative period. Satisfaction was good and there was no association with the characteristics of nurses’ attention. It is recommended to improve these predictors to increase the safety of surgical patients.

## Abstract (pt)

**Objetivo:**

investigar a segurança e a satisfação de pacientes e sua relação com os cuidados dos enfermeiros no perioperatório.

**Método:**

estudo multinível, transversal, correlacional, realizado com 105 enfermeiros da área cirúrgica e 150 pacientes operados em um hospital terciário espanhol. Para os enfermeiros foram coletadas as variáveis sociodemográficas, a percepção do ambiente de trabalho, o desgaste profissional e a satisfação no trabalho. Para os pacientes, a segurança de eventos adversos e nível de satisfação, por meio de registros e questionários. Utilizou-se análise multinível, univariada e multivariada.

**Resultados:**

satisfação no trabalho, compromisso profissional e participação em questões hospitalares foram preditores negativos para eventos adversos relacionados ao paciente, enquanto o cuidado do enfermeiro no pós-operatório foi um preditor positivo.

**Conclusão:**

observa-se aumento dos eventos adversos quando os enfermeiros têm insatisfação no trabalho, menor compromisso profissional e baixa disponibilidade para participar nos assuntos de sua unidade. Por outro lado, os eventos adversos diminuem quando os enfermeiros realizam os cuidados no pós-operatório. A satisfação foi boa e não houve associação com as características do cuidado dos enfermeiros. Recomenda-se melhorar esses preditores para aumentar a segurança de pacientes cirúrgicos.

## Abstract (es)

**Objetivo:**

investigar la seguridad y la satisfacción de los pacientes y su relación con el cuidado de los enfermeros en el perioperatorio.

**Método:**

estudio multinivel, transversal, correlacional, realizado con 105 enfermeros del área quirúrgica y 150 pacientes operados en un hospital terciario español. Para los enfermeros fueron recolectadas las variables sociodemográficas, la percepción del ambiente de trabajo, el desgaste profesional y la satisfacción en el trabajo. Para los pacientes, la seguridad de eventos adversos y el nivel de satisfacción, mediante la aplicación de cuestionarios. Se utilizó análisis univariante y multivariante.

**Resultados:**

satisfacción en el trabajo, compromiso profesional y participación en cuestiones hospitalarias fueron predictores negativos para eventos adversos relacionados al paciente, mientras que el cuidado del enfermero en el postoperatorio fue un predictor positivo.

**Conclusión:**

se observa aumento de los eventos adversos cuando los enfermeros tienen insatisfacción en el trabajo, menor compromiso profesional y baja disponibilidad para participar en los asuntos de su unidad. Por otro lado, los eventos adversos disminuyen cuando los enfermeros realizan los cuidados en el postoperatorio. La satisfacción fue buena y no hubo asociación con las características de la atención de los enfermeros. Se recomienda mejorar estos predictores para aumentar la seguridad de los pacientes quirúrgicos.

## Introduction

The goal of a healthcare system is to ensure safe and quality health care. In this context, patient safety is a major concern today. In the context of the Patient Safety Program, the World Health Organization (WHO), develops programs that address the different risks to patients around the world^(^ ^1^ ^)^. In Spain, the Ministry of Health has placed patient safety as one of the key elements to improve the quality of care, according to the 2015-2020 Patient Safety Strategy. This guide describes details of the recommendations applicable to the different areas of care and to all professionals in the health team^(^ ^2^ ^)^. Nurses stand out as members of health care teams because they play a key role in direct patient care and an important role in the detection and prevention of adverse events (AE). An adverse event corresponds to any unintentional injury or complication resulting from healthcare. AE are indicators of patient safety and quality of care^(^ ^3^ ^)^. In the nursing field, AE are called nursing-sensitive outcomes^(^ ^4^ ^)^. The most common indicators of AE related to nursing care are errors in medication administration, falls, pressure ulcers, resuscitation failures, rescue failures, nosocomial infections, and follow-up of procedures^(^ ^5^ ^)^.

On the other hand, patient satisfaction about the care received is considered an indicator of quality^(^ ^6^ ^)^. The main causes of AE in healthcare are related to human factors, such as professional competence to assess risks, and also factors related to the system, such as conditions and characteristics of the environment in which the nurses develop their work^(^ ^7^ ^)^. The personal and environmental characteristics of their practice are critical predictors of patient care quality^(^ ^8^ ^)^. The association between the characteristics of the nurses’ work environment and higher levels of training and personal endowment creates a better working environment and promote favorable outcomes in patient health, even with respect to mortality^(^ ^9^ ^)^. Other factors in the work environment have been associated to the quality and safety of patient care, including the physical environment, working hours, and the extent of exhaustion of nursing professionals^(^ ^10^ ^)^.

Most investigations have been carried out at the hospital level^(^ ^11^ ^)^. However, research in complex areas such as in the surgery context, is very scarce and yet a very important focus due to the volume of interventions performed worldwide each year (234 million). Surgical care leads to a considerable risk of AE that contributes to increasing the burden of morbidity. However, 50% of the complications that arise can be avoided through strategies such as “safe surgery saves lives”^(^ ^12^ ^)^. To avoid complications and AE in the surgical area, nursing interventions should cover the entire perioperative period, i.e. before, during, and after surgery^(^ ^13^ ^)^. In this sense, the impact of interventions provided by perioperative nurses on patient health outcomes, although relatively little known, seems to be of great importance. Some authors have investigated the relationship between the nursing team and complications in surgical patients^(^ ^14^ ^)^, as well as the phenomenon of Burnout in the surgical center^(^ ^15^ ^)^. However, the relationship between perioperative nursing and patient outcomes has not been studied. For this reason, the present research raises questions about the impact of perioperative nurses and of the environment of their practice on the outcomes of surgical patients? This study has therefore the objective to investigate the safety and satisfaction of patients and their relationship with nurse's care in the perioperative period.

## Method

This work applied a cross-sectional and correlational design with two convenience samples. The first includes nurses from the surgical area, n = 105.

All the nurses who worked in the perioperative, transoperative and postoperative unit of the surgical area were contacted to participate. Nurses who were absent due to vacations or sick leave during the study period were not included. The second sample, n = 150, was composed of patients operated in different specialties: general surgery, orthopedic surgery and traumatology, thoracic surgery, gynecological surgery, neurosurgery, and plastic surgery. The patients excluded from the study were those under 18 years of age, with cognitive deficits, who had undergone endotracheal intubation for more than 48 hours, or those who had been discharged within 24 hours after the intervention. The sample size was calculated considering a confidence interval (CI) of 95 under the hypothesis of maximum intermissions (p = q = 50%) and a margin of error of ±1.19% in the sample of nurses and ±of 1.13% in the sample of patients. Data were collected during the period 2014-2015 at the Hospital de la Santa Creu i Sant Pau, Barcelona. Spain

The study combines data collected from the perioperative nursing unit at the level of individual nurses and at the level of patient through various data sources. The first source was a questionnaire applied to nurses to collect information on the characteristics of the organization and of the perioperative unit (nurses’ practice environment), and on sociodemographic (age and sex) and work (academic training, work experience, type of contract, job satisfaction, intention to leave the hospital, and burnout) aspects. The second source came from the patient satisfaction questionnaire, and the third source was patient data on management, adverse event reports, mortality, and clinical outcomes.

The Spanish version of the Practice Environment Scale of the Nursing Work Index (PES-NWI), which presented Cronbach's alpha values of 0.90 (95% CI: 0.87-0.93), was used to measure the environment or the practice environment of the nurses^(^ ^16^ ^)^. The index is composed of 31 items and is structured in five factors: (1) personal and resources; (2) working relationships between nurses and physicians; (3) leadership and support from supervisors; (4) nursing bases for quality care; and (5) nurses’ participation in hospital matters. The professionals had to assess their relevance in a Likert-type scale varying from 1 to 4 (1 = totally disagree, 2 = disagree, 3 = agree, and 4 = totally agree). Once the evaluations were obtained, the work environment was classified as favorable when presenting 4 or 5 factors with an average score higher than 2.5, mixed in the case of having 2 or 3 factors, and unfavorable in case of having 1 or no factor.

The Spanish version of the Maslach Burnout Inventory (MBI)^(^ ^17^ ^)^ was used to the measure professional burnout of the nursing staff. MBI is the most frequently used tool to measure burnout caused by work and consists of three dimensions: emotional exhaustion (EE), depersonalization (DP) and personal accomplishment (PA). The inventory contains 22 items measured on a Likert-type scale with scores from 1 to 7 points (from «never» to «every day»). The MBI established that the three dimensions are categorized into three groups each (low, medium and high) according to the following values: EE: low ≤ 18, medium [19-26], high ≥ 27; DP: low ≤ 5, medium [6-9], high ≥ 10; PA: low ≥ 40, medium [39-34], high ≤ 33. The reliability and validity of this tool, obtained in another study, demonstrated its applicability^(^ ^18^ ^)^.

To measure the nurses’ satisfaction, we followed the methodology used in the RN4CAST project. A single question with Likert-type scale (1 “Very dissatisfied” to 4 “Very satisfied”) was used to evaluate satisfaction with the current work (coefficient of reliability 0.7). The satisfaction questionnaire was also applied to nine specific aspects of the work: flexibility of time, professional development, autonomy at work, salary, training, vacations, commitment, sick leave, and permission to study^(^ ^19^ ^–^ ^20^ ^)^. As to patients, data on sociodemographic aspects (age and sex), the specialty of the surgery to which they were submitted, the presence of comorbidities, and the length of hospital stay were collected. Patient safety outcomes were analyzed by assessing the presence of adverse events, including mortality and rescue failure. The indicators of EA of the 150 patients were collected from records of adverse events reported in the surgical area and in medical records. The criteria and data sources for each outcome were based on the SENECA100 model: pressure injuries, nosocomial infections, phlebitis, medication-related AE, postoperative complications and pain. This model was used in another study at the national level^(^ ^21^ ^)^, which coincided with reliable and valid indicators in international studies^(^ ^22^ ^)^. For this study, the AE were recoded in a dichotomous variable (absence/presence of AE) to relate them to the characteristics of the nurses.

LaMonica-Oberst Patient Satisfaction Scale 12 (LOPSS-12) adapted in Spanish^(^ ^23^ ^)^, with Likert-type scale responses ranging from 1 (totally agree) to 5 (totally disagree) was used to analyze the satisfaction of patients with nursing care. All elements are related to the care provided by the nursing staff, for example: “They help me understand my illness”. The original scale was structured in two satisfaction factors: the positive and the negative factor, which were difficult to measure. For this reason, we chose to recodify it in one direction, calculating the arithmetic mean of the responses given to the 12 items: the higher the score obtained, the higher the degree of patient satisfaction, as in another study^(^ ^24^ ^)^. The internal consistency of the LOPPS questionnaire was 0.81 (Cronbach's alpha). In addition, patients were asked if they would recommend the hospital to others. The questionnaires were self-completed, after signing the Informed Consent Form.

Regarding the treatment and analysis of data, the descriptive analysis of the characteristics of nurses and patients was done using absolute frequencies and percentages for the qualitative variables, and means and standard deviation (SD) for the quantitative variables. Considering that there were set of patients assisted by the same nurse (105 nurses for 150 patients), multiple-level analyses were performed incorporating the hierarchical structure of the data, that is, patients nested within nurses. The multilevel full regression model assumes a set of hierarchical data with the dependent variable (presence/absence of AE) measured at the lowest level (patients) and the explanatory variables that exist at both levels. In the present study, the efficient way to correct the variable nurse that assists the patient is to use the multilevel analysis, that is, the nurse variable as the second level. Observations made at the level of patient are nested at the level of nurse.

Taking into account this hierarchical structure of the data, the following analysis were made: estimation of the mean in different variables through the models that include the variable of random effects and variable of fixed effects. A univariate analysis was performed between each of the independent variables (fixed effects) and the scores of the dependent variables through simple multilevel linear regression models. In turn, a multivariate analysis was made using multilevel multiple linear regression models for the independent variables (fixed effects) that were taken to the multivariate models that were those that obtained a level of significance p < 0.001 in the univariate analysis. A hierarchical structure of the data was established and the variables were inserted in the model to estimate the effect of the two levels, where the individual level 1 or base level is the patient, and the level 2 or the higher level is the group of nurses in the surgical area. Thus, there were 150 surgical patients (level 1) attended in the surgical area by the group of 105 nurses (level 2). In our models, the response or dependent variables were AE within the 30 days after the intervention, on the one hand (considered dichotomized, i.e. presence/absence), and satisfaction of surgical patients on the other. The variables of random and fixed effects were those related to the characteristics of patients and nurses. Each of the 150 patients was treated in the surgical area by more than one nursing professional. Our data indicate that at least five and at most 12 professionals assisted each patient. The group of 105 nurses from the surgical area was included because they assisted the 150 patients submitted to surgery. The most usual number of patients assisted by a nursing professional was four (14 times), but it was also noticed that there were professionals who observed two patients (11 times), eight patients (10 times), and 12 patients (10 times). Each of the 150 patients assisted by the group of 105 nurses generated a database of 1422 records. This, therefore, is the valid N of the analysis. This N is highly representative (95% confidence, p = q = 50%) with a margin of error of 0.37%.

In the first part of the statistical analyses, a univariate analysis was performed with the objective of predicting the appearance of AE based on the independent variables of the patients and the variables of nurses who assisted such patients. Then, the multivariate and multilevel analysis procedure was applied to determine the factors of patients and nurses that were significant predictors of the presence variable of AE. To this end, only those factors that were statistically significant at least for p < 0.001 in the previous univariate analysis were considered. For the multivariate analysis, null model tests determined whether a predictive model of multiple levels was possible^(^ ^25^ ^)^. The null model for baseline analysis (patients) presented a statistical value of Chi^2^== 1718.66, with p < 0.001 model were performed; highly significant; and the null model for the higher level (nurses) had a value of Chi^2^ = 161.52 with p 0.001; both highly significant. Therefore, a multilevel predictive model was made, based on the variables of the patients and on the variables of the nurses who assisted them. Significance was considered when the p value was lower than 5% (p < 0.05). However, given the high N, high significance was only considered when the variables reached significance (p 0.001).

The statistical package STATA Statistics Data Analysis v.12.0 was used for the multilevel analysis. For the rest of the analyses, the statistical application IBM SPSS Statistics v-22.0 was used.

International ethical recommendations for medical research in human subjects were followed closely in this study. The study was approved by the Ethics Committee of the Hospital de la Santa Creu i Sant Pau (CEIC Code: 42/2014). The security and confidentiality of the study data were guaranteed in accordance with the provisions of Organic Law 15/1999 of 13 December on the Protection of Personal Data.

## Results

Description of the results concerning nurses: 105 questionnaires filled out by the perioperative nurses were collected. A total of 91.5% (96) of the nursing professionals were women. The mean age of the women was 44.0 years (standard deviation of 11.90), higher than that of men who was 36.7 years (10.26), the most significant difference (p = 0.51). The average professional experience of the professionals was 21.6 years (SD 12.13) in total and 14.0 years (SD 11.14) in the current working environment. With respect to training, 98.4% (103) of the nurses had specialization, among them 33.4% (35) had master's degree and 66.6% (70) had post-graduate degree. Description of patient outcomes: 150 surgical patients were included until the sample size was reached. A total of 45.3% (68) underwent general surgery, 19.3% (29) orthopedic surgery, 9.3% (14) thoracic surgery, 8% (12) vascular surgery, 10% (15) gynecology, 6.7% (10) neurosurgery, and 1.3% (2) plastic surgery. The study had 77 men (51.3%) and 73 women (48.6%), with a mean age of 63.6 years (SD 16.05). The discharge destination was the patient's home in the case of 94.5% (141.75) of the cases, and the mean time of hospital stay was 24.9 hours (SD 3.7). As for comorbidities, 46% (69) of the patients presented some type of comorbidity.

Regarding AE, 38% (57) of the surgical patients in the study presented some type of AE during the surgical process, from the time of admission up to 30 days after the intervention. The most frequent AE was the presence of pain, present in 23.3% of the cases (35). Postoperative complications included reintervention or bleeding in 8% (12) patients, wound infection in 6.4% (10), followed by position or pressure injuries in 3.3% (5), urinary infection in 2% (3), respiratory infection in 1.3% (2) and medication error in 0.6% (1). There were no other types of AE in these patients.

The results for the variables of patient characteristics (predictive factors) of the presence/absence of adverse events within 30 days postintervention are presented in Table 1.

**Table 1:** Univariate multilevel analysis. Variables of patients’ characteristics and presence/absence of AE* in patients within 30 days post-intervention (N = 1422) Barcelona, Spain 2014-2015

Patient Variables		Presence of AE*%	Absence of AE*%	p†
Sex	Female	38.0	62.0	0.408
	Male	40.1	59.9	
Comorbidity	Yes	43.5	56.6	<0.001†
	No	35.2	64.8	
Expertise	General surgery	41.8	58.2	<0.001†
	Traumatology	41.2	58.8	
	Gynecology	31.7	68.3	
	Thoracic surgery	28.6	71.4	
	Vascular surgery	33.3	66.7	
	Neurosurgery	52.4	47.6	
	Plastic surgery	0	100	
Age *(years)*	Mean (SD)‡	63.5(14.33)	63.3(17.17)	0.900
Stay *(hours)*	Mean (SD)‡	25.04(3.73)	24.8 (4.0)	0.321

* AE: Adverse Event,

† p: p-value significance,

‡ SD: Standard deviation.

The association between the existence of comorbidity and the appearance of AE in operated patients was highly significant. The relationship between the type of surgical specialty and the presence/absence of AE was also significant. The appearance of AE was more frequent in cases of neurosurgery (52.4%) than in the rest of the specialties (between 28.6% in thoracic surgery and 41.8% in general surgery). No association was found among the other analyzed variables.

In the second analysis, an association was made between the variables characteristics of the nursing work environment and presence/absence of AE within 30 days post-intervention. (Table 2)

**Table 2:** Univariate multilevel analysis. Significance in the relation of variables with the nurses’ characteristics and presence/absence of AE* in patients within 30 days post-intervention (N = 1422) Barcelona, Spain, 2014-2015

Nurses’ variables		Presence of AE*	Absence of AE*	p†
Age *(years)*	Mean (SD)‡	47.21 (12.23)	45.23 (13.09)	0.004
Nurse - Pre-operative	Yes No	27.1 % 40.8 %	73 % 59.2%	<0.001†
Nurse - Post-operative	Yes No	44.7 % 34.8 %	55% 65.2 %	<0.001†
Type of contract	Eventual	40.4 %	59.6%	0.004
PES-NWI§ factor1	Mean (SD)‡	2.08 (0.62)	2.27 (0.57)	<0.001†
PES-NWI§ factor2	Mean (SD)‡	2.28 (0.78)	2.50 (0.67)	<0.001†
PES-NWI§ factor3	Mean (SD)‡	2.20 (0.79)	2.55 (0.66)	<0.001†
PES-NWI§ factor4	Mean (SD)‡	2.53 (0.58)	2.80 (0.55)	<0.001†
PES-NWI§ factor5	Mean (SD)‡	1.91 (0.46)	2.16 (0.48)	<0.001†
MBI‖ Emotional Exhaustion	Mean (SD)‡	1.92 (0.87)	1.56 (0.81)	<0.001†
Current satisfaction	Mean (SD)‡	2.10 (0.35)	2.24 (0.47)	<0.001†
Flexibility of time	Mean (SD)‡	2.42 (0.65)	2.59 (0.74)	<0.001†
Professional development	Mean (SD)‡	2.15 (0.56)	2.24 (0.69)	<0.001†
Autonomy at work	Mean (SD)‡	2.15 (0.74)	2.41 (0.81)	<0.001†
Salary	Mean (SD)‡	2.04 (0.24)	2.02 (0.22)	0.351
Training	Mean (SD)‡	1.99 (0.21)	2.04 (0.31)	<0.001†
Vacations	Mean (SD)‡	2.10 (0.35)	2.24 0.47)	< 0.001†
Sick leave	Mean (SD)‡	2.04 (0.26)	2.08 (0.31)	0.042
Permission to study	Mean (SD)‡	2.13 (0.43)	2.22 (0.50)	< 0.001†
Professional commitment	Mean (SD)‡	3.37 (1.20)	3.92 (1.14)	< 0.001†

* AE: Adverse Event;

† p: p-value significance;

‡ SD: standard deviation;

§ PES-NWI: Scale of the nurse's practice environment;

‖ MBI: Maslach Burnout Inventory.

The frequency of onset of AE in patients was significantly lower when nurses assisted them in the preoperative period (27.1% vs. 40.8%). On the other hand, a higher frequency of patients with AE was significantly associated with less care of nurses in the postoperative unit (44.7% vs. 34.8%). The mean of the five PES-NWI factors was also significantly lower in nurses who treated patients with AE. Of the three dimensions of the MBI, there was a greater emotional exhaustion of nurses assisting patients with some AE. Finally, all variables related to job satisfaction, with the exception of salary, obtained lower scores in nurses who assisted patients with AE.

After this, a multivariate analysis was performed. The coefficients (r) are presented in the univariate way for all the independent variables analyzed and adjusted for those variables that were included in the final multivariate model (Table 3).

**Table 3:** Multivariate multilevel analysis. Significance of predictive factors (nurses and patients) on the presence of adverse events within 30 days after the intervention (N = 1422). Barcelona, Spain 2014-2015

Predictors (fixed effects factors)	Unadjusted values			Adjusted values		
	Coe*	S.E†	p-value‡	Coe*	S.E†	p‡
Age	0.250	0.232	0.325	--	--	--
Preoperative nurse	-0.481	0.467	0.302	--	--	--
Postoperative nurse	0.903	0.248	<0.001‡	0.710	0.217	<0.001‡
Type of eventual contract	-0.722	0.684	0.295	--	--	--
PESNWI§ Fator1	-0.367	0.183	0.044	-0.124	0.175	0.477
PESNWI§ Factor2	-0.224	0.175	0.200	--	--	--
PESNWI§ Factor3	-0.527	0.157	<0.001‡	-0.014	0.198	0.942
PESNWI§ Factor4	-0.504	0.217	0.020	0.254	0.254	0.319
PESNWI§ Factor5	-0.888	0.252	<0.001‡	-0.640	0.235	0.007
MBI‖ Exhaustion	0.511	0.140	<0.001‡	0.152	0.135	0.260
Current satisfaction	-0.656	0.289	0.023	-0.780	0.270	0.004
Flexibility of time	-0.377	0.173	0.030	-0.261	0.155	0.094
Professional development	-0.348	0.156	0.025	0.215	0.144	0.136
Autonomy at work	-0.212	0.203	0.296	--	--	--
Training	-0.518	0.505	0.305	--	--	--
Vacations	-0.448	0.235	0.057	--	--	--
Sick leave	0.695	0.361	0.054	--	--	--
Permission to study	1.136	0.805	0.158	--	--	--
Professional commitment	0.392	0.103	<0.001‡	-0.280	0.098	0.004
Patient Comorbidity	0.274	0.129	0.033	0.230	0.128	0.074
Neurosurgery Patient	0.946	0.242	<0.001‡	0.880	0.240	<0.01

* Coe: Regression coefficient;

† S.E: Standard error;

‡ p: p-value: significance;

§ PES-NWI: Scale of nurses’ practice environment;

‖ MBI: Maslach Burnout Inventory.

The final result presented four significant factors: Participation in hospital matters (r = −0.640, p = 0.007); Job satisfaction (r = −0.780, p = 0.004) and professional commitment (r = −0.280; p = 0.004) resulted to be negative predictive factors. On the other hand, care from nurses in the postoperative period (r = 0.710, p = 0.001) was a positive predictive factor for the presence of AE in the patients. For the significant variables, the percentages were: Participation in hospital matters 4.1%; job satisfaction 2.6%; professional commitment 1.7%; and nurses in the postoperative period 1.2%. The complete model reached an explained variance of 14.6%.

For the analysis of patient satisfaction with nursing care, the dependent variable *Total Satisfaction* was previously calculated based on the patients’ responses on the LOPPS scale 12. They were recoded in the same direction and the highest score corresponded to the highest patient satisfaction. The dependent variable of *total patient satisfaction* was calculated as the arithmetic mean of the 12 questions. This variable had a nearly normal distribution, with a good degree of symmetry, but with a higher height (kurtosis) in the central values. The mean of this variable was 3.66 (SD 0.37) within a range between 2.75 and 5.00 (median 3.58).

In general, the degree of satisfaction was high in all the questions. The average values were above four points; the most valued questions were the 11 “carry out their work with responsibility” and 2 “interest in listening”. And the most valued questions were the 8 “they show empathy” and the 7 “they give useful advice”.

In the analysis of the association of the variables patients’ characteristics with *total patient satisfaction*, statistical significance (p < 0.001) was obtained for all, except for the patient age. However, the Pearson coefficient (r) values of the quantitative and categorical factors indicated that, although the associations were significant due to the large sample size, the intensity of the association was very low. The results for the variables (predictive factors) of the patients are summarized in Table 4.

**Table 4:** Associative Analysis. Relationship between variables of the patients’ characteristic and total patient satisfaction (mean of the items of the LOPSS 12) (N = 1422). Barcelona, Spain 2014-2015

Patient variable		Satisfacción total	P
		(Media 3.66; DE* 0.37)	
Sex	Female	3.68 (0.39)	0.008†
	Male	3.63 (0.35)	
Comorbidity	Yes	3.63 (0.42)	0.007†
	No	3.68 (0.33)	
Specialty of the surgery	General surgery	3.65	
	Traumatology	3.66	
	Gynecological surgery	3.64	
	Surgery. Thoracic	3.68	
	Vascular surgery	3.57	
	Neurosurgery	3.82	
	Plastic surgery	3.39	<0.001‡
Age (years)		r −0.050§	0.057‖
Length of stay (hours)		r −0.140§	<0.001‖

* SD: Standard deviation;

† p-value: Student t test;

‡ P value: chi-square test;

§ r: Pearson's correlation coefficient;

‖ Z normal.

No variable was found to be significantly associated (p > 0.05) when the variables of nurses’ characteristic were crossed with *total patient satisfaction*. Consequently, none of the nurses’ characteristics was able to effectively predict patient satisfaction, as described in the table below (Table 5).

**Table 5:** Associative Analysis. Relationship between variables characteristic of nurses and total patient satisfaction (N = 1422) Barcelona. Spain 2014-2015

Nurses’ variables		Satisfacción total	*Contrast test*
		(Mean 3.66, SD* 0.37)	p-value
Sex	Fémale	3.66 (0.37)	0.687†
	Male	3.67 (0.41)	
Postgraduate/master's degree	Yes/No	3.66 (0.37)	0.855†
Transoperative nurse	Yes/No	3.66 (0.40)	0.826†
Preoperative nurse	Yes/No	3.66 (0.37)	0.213†
Postoperative nurse	Yes/No	3.66 (0.37)	0.908†
Contract Type	Permanent/Eventual	3.66 (0.37)	0.675†
Age		-0,006‡	0.812^§^
Work experience		0.001‡	0.982^§^
Current work experience		0.020‡	0.441^§^
PES-NWI‖ factor1		0.004‡	0.889^§^
PES-NWI‖ factor2		-0.025‡	0.339^§^
PES-NWI‖ factor3		-0.038‡	0.148^§^
PES-NWI‖ factor4		0.002‡	0.938^§^
PES-NWI‖ factor5		-0.013‡	0.627^§^
MBI¶ Depersonalization		0.015‡	0.581^§^
MBI¶ Personal accomplishment		0.006‡	0.824^§^
Satisfaction in the current job		0.003‡	0.909^§^
Flexibility of time		-0.029‡	0.276^§^
Professional development		-0.044‡	0.100^§^
Autonomy at work		-0.010‡	0.708^§^
Salary		0.003‡	0.906^§^
Training		-0.012‡	0.649^§^
Vacations		-0.029‡	0.278^§^
Sick leave		-0.013‡	0.630^§^
Permission to study		-0.026‡	0.328^§^
Professional commitment		-0.034‡	0.199^§^

* SD: Standard deviation;

† p-value of Student t test;

‡ r: Pearson's correlation coefficient;

‖ PES-NWI: Nursing Practice Environment Scale

¶ MBI: Burnout Maslach Inventory

The results showed no relations between the variables. In order to propose a multilevel analysis, there must be a correlation between the variables. The study led to the conclusion that it makes no sense to consider a multilevel analysis since the only factors associated with patient satisfaction are variables of patients’ characteristics alone (although their limited effect was mentioned despite their significance). We also analyzed the possibility of running a multiple regression model with the patients’ predictors that were significant in Table 4. However, the quality was very low because the total predictive capacity was only 2.2%, totally irrelevant from the point of view of its effectiveness.

Regarding the question made to the patients about whether they would recommend the hospital to other patients, 91.3% (119) said they would do so. Thus, only 8.7% (11) would not recommend.

## Discussion

In this study, the multilevel methodology was used to investigate the safety and satisfaction of patients and their relationship with nurse's care in the perioperative period. The results were collected, as in other studies, analyzing the presence of adverse events and the patients’ perception about nursing care^(^ ^26^ ^–^ ^27^ ^)^, which may have positive and negative effects. In relation to the nursing team, the main associations with AE are the nurses’ practice environment, emotional exhaustion, job satisfaction, years of experience, and type of contract. Regarding patients, it is worth mentioning the presence of comorbidity and type of surgery (neurosurgery). Working conditions, as a result of increased surgical activity, cause a heavy workload. Problems related to the maintenance of personnel, such as personnel changes and excessive use of temporary staff due to the generational change in our perioperative area influenced these associations. We agree that these problems are risk factors for patient safety^(^ ^22^ ^,^ ^28^ ^–^ ^29^ ^)^. Confirming the present results, the predictors of AE are job satisfaction, participation in hospital matters, professional commitments, and postoperative care, coinciding with other studies^(^ ^27^ ^,^ ^30^ ^–^ ^31^ ^)^. The importance of having a positive practice environment for the work of nurses was clear. Such aspect increases the job satisfaction, commitment, and retention of nurses and the best outcomes for patients. Research in magnetic hospitals has extensively documented the impact of nursing care on both nurse and patient outcomes^(^ ^32^ ^)^. The record of the reported events was 38%. It is a value that is not high in relation to other investigations^(^ ^33^ ^)^, although it includes the presence of all the AE attributable to patients during the perioperative period. However, the analysis of six or less AE is more usual in other studies^(^ ^34^ ^–^ ^35^ ^)^. There is another difference between our study and the others in which there was no mortality^(^ ^9^ ^,^ ^36^ ^)^. The most reported AE was the presence of pain, followed by postoperative complications (bleeding and wound infection). This is similar to national^(^ ^21^ ^,^ ^37^ ^)^ and international^(^ ^38^ ^)^ studies and suggest that efficient measures should be taken and safe practices applied^(^ ^12^ ^)^. It is important to note that most AE, such as pain, can be prevented or eliminated if detected early.

Regarding patient satisfaction, the characteristics of the nurses did not present a significant association with it. The current findings may have been influenced by confounding factors that were not assessed, such as other individual or organizational characteristics that were not considered. However, the behaviors of nurses during perioperative care were positively evaluated by the patients^(^ ^24^ ^)^. This is a very positive aspect because the patient's experience results from the actual quality of care and from their perception^(^ ^39^ ^)^. One of the most important results was that the vast majority of patients (119), i.e. 91.3%, answered that they would recommend the hospital to others (for example, friends or relatives). Patients had positive perceptions of the nursing care and a greater likelihood of satisfaction with general care. As different studies suggest that satisfaction with the care provided represents an important part of the quality of hospital care, the present findings are a good result for perioperative nurses and for the organization^(^ ^40^ ^–^ ^41^ ^)^.

The main limitation of the study is that data collection was restricted to a single hospital, convenience samples were used, and studies in the surgical field to allow a comparison are missing. Furthermore, most studies on patient outcomes did not examine all AE; they present rather an incomplete picture of safety. Differences in the methodology of the studies make it difficult to compare the outcomes. Despite these limitations, there are no recent studies examining the impact of perioperative nurses on the safety and satisfaction of surgical patients. For the first time, the effect of perioperative nursing care in the unit of work was related to safety outcomes of surgical patients. In fact, we related the presence of AE and complications with the care provided by nurses. The multilevel analysis allowed to incorporate in the same model the independent variables belonging to different levels, the variables of individual patients (first level) and the variables of nurses and of the unit (second level). This study contributed to the identification of areas of improvement in the context of safety culture. It also showed the impact that different aspects such as job satisfaction, professional commitment, and work the environment have over the quality of care.

## Conclusion

Job satisfaction, professional commitment, and participation in hospital matters were negative predictors of adverse events in patients, especially pain and postoperative bleeding complications. In turn, care from postoperative nurses acted as a positive predictor. If nurses are dissatisfied at work, have less professional commitment, and have a low perception of participation in matters taking place in their unit, the adverse events in the patients cared for by them increase. On the other hand, nurses who perform postoperative care help to decrease them. There was no association with satisfaction outcomes. Therefore, perioperative nurses have an impact on safety outcomes, but not on satisfaction of surgical patients. The key to ensuring the quality of care for surgical patient is a positive work environment that promotes job satisfaction, professional commitment, quality of nursing care throughout the perioperative process, and active participation of the nurse in the unit and hospital matters. It is recommended that administrators and managers of the surgical field implement strategies to improve these aspects so as to improve safety. Researchers are encouraged to conduct further research in this field of nursing practice with comparable samples in perioperative units.
